# Brown Adipose Tissue Activation Is Involved in Atherosclerosis of ApoE^−/−^ Mice Induced by Chronic Intermittent Hypoxia

**DOI:** 10.3389/fcvm.2021.751519

**Published:** 2021-10-26

**Authors:** Yue Wang, Hong-feng Jiang, Bei-bei Liu, Lei-lei Chen, Yue Wang, Xin-yan Liu, Min Suo, Xiao-fan Wu

**Affiliations:** ^1^Department of Cardiology, Beijing Anzhen Hospital, Capital Medical University, Beijing, China; ^2^Key Laboratory of Remodeling-Related Cardiovascular Diseases of Ministry of Education, Beijing Collaborative Innovation Center for Cardiovascular Disorders, Beijing Institute of Heart Lung and Blood Vessel Diseases, Beijing Anzhen Hospital, Capital Medical University, Beijing, China; ^3^Beijing Institute of Heart, Lung, and Blood Vessel Disease, Beijing Anzhen Hospital, Capital Medical University, Beijing, China; ^4^Center for Coronary Artery Disease, Beijing Anzhen Hospital, Capital Medical University, Beijing, China

**Keywords:** chronic intermittent hypoxia, brown adipose tissue (BAT), uncoupling protein 1 (UCP1), atherosclerosis, obstructive sleep apnea

## Abstract

**Background:** Obstructive sleep apnea is an atherogenesis factor of which chronic intermittent hypoxia is a prominent feature. Chronic intermittent hypoxia (CIH) exposure can sufficiently activate the sympathetic system, which acts on the β3 adrenergic receptors of brown adipose tissue (BAT). However, the activity of BAT and its function in CIH-induced atherosclerosis have not been fully elucidated.

**Methods:** This study involved ApoE^−/−^ mice which were fed with a high-fat diet for 12 weeks and grouped into control and CIH group. During the last 8 weeks, mice in the CIH group were housed in cages to deliver CIH (12 h per day, cyclic inspiratory oxygen fraction 5–20.9%, 180 s cycle). Atherosclerotic plaques were evaluated by Oil Red O, hematoxylin and eosin, Masson staining, and immunohistochemistry. Afterward, we conducted immunohistochemistry, western blotting, and qRT-PCR of uncoupling protein 1 (UCP1) to investigate the activation of BAT. The level of serum total cholesterol (TC), triglyceride, low-density lipoprotein cholesterol (LDL-c), high-density lipoprotein cholesterol (HDL-c), and free fatty acid (FFA) were measured. Finally, RNA-Sequencing was deployed to explore the differentially expressed genes (DEGs) and their enriched pathways between control and CIH groups.

**Results:** Chronic intermittent hypoxia exposure promoted atherosclerotic plaque area with increasing CD68, α-SMA, and collagen in plaques. BAT activation was presented during CIH exposure with UCP1 up-regulated. Serum TC, triglyceride, LDL-c, and FFA were increased accompanied by BAT activation. HDL-c was decreased. Mechanistically, 43 lipolysis and lipid metabolism-associated mRNA showed different expression profiling between the groups. Calcium, MAPK, and adrenergic signaling pathway included the most gene number among the significantly enriched pathways.

**Conclusion:** This study first demonstrated that BAT activation is involved in the progression of CIH-induced atherosclerosis, possibly by stimulating lipolysis.

## Introduction

Atherosclerosis is a leading cause of vascular disease worldwide. Its major clinical manifestation, which is cardiovascular disease (CVD), remains the leading cause of mortality in the world (1). Recent studies stated that obstructive sleep apnea (OSA), which affects 34% of men and 17% of women and is largely undiagnosed (2), is a modifiable CVD risk factor (3, 4). Chronic intermittent hypoxia (CIH) is a prominent feature of OSA pathophysiology and could be a major mechanism linking OSA to arteriosclerosis (3). Abundant evidence from animal studies has linked atherosclerosis to CIH (5–7). Despite this long-known linkage between CIH and high atherosclerosis risk, mechanisms underlying CIH-induced atherosclerosis have not been fully elucidated, although NF-κB pathway, oxidative stress, and neuroendocrine disorders have been claimed to be associated with atherosclerosis incidence (8, 9).

In rodents, CIH exposure can sufficiently activate the sympathetic system, leading to an increased level of norepinephrine (10, 11), which acts on the β3 adrenergic receptors of brown adipose tissue (BAT) (12). Brown adipocytes contain many mitochondria that have uncoupling protein 1 (UCP1) in their inner membrane (13). UCP1, which is only expressed in brown adipocytes, uncouples the respiratory chain from oxidative phosphorylation yielding a high oxidation rate and enabling the cell to use metabolic energy to provide heat (14). BAT is found firstly as the main site of adaptive thermogenesis. In rat and mouse models, BAT generates heat to enable the organism to adapt to a cold environment (15, 16). While initially believed to be of relevance only in human newborns and infants, research during recent years provided unequivocal evidence of active BAT in human adults (17). Moreover, it has become clear that BAT plays an important role in insulin sensitivity and metabolism in rodents and humans (18–20). These data all indicated that BAT plays an important role in the metabolism of both rodents and humans. However, it remains unclear whether BAT takes part in CIH-induced atherosclerosis, as a kind of metabolic disorder.

Brown adipose tissue activation has been reported in ApoE^−/−^ mice fed with a high-fat diet at 23°C (21). It has also been reported that cold exposure promotes atherosclerotic plaque growth and instability *via* UCP1-dependent lipolysis in ApoE^−/−^ mice and Ldlr^−/−^ mice at 4°C (22). Nevertheless, whether BAT is involved in metabolic dysregulation in CIH-induced atherosclerosis has not been explored. In this study, we investigated the impact of CIH-induced BAT activation on atherosclerotic plaque formation in ApoE^−/−^ mice. We reported our findings that CIH exposure significantly accelerated the atherosclerotic plaque growth in ApoE^−/−^ mice by the possible mechanism of accelerated UCP1- mediated lipolysis.

## Methods

### Animals

Male homozygous ApoE^−/−^ mice (C57BL/6J background) were purchased from the Hua Fukang Bioscience Company (Beijing, China). All the mice were obtained at 7 weeks of age, housed in a specific pathogen-free facility under a 12/12 h light-dark cycle at 25°C, acclimated for a week prior to the study, and fed with a high-fat diet (0.15% cholesterol and 21% fat, 4 kcal/g) during the experiment. The mice had unrestricted access to food and tap water. All experimental protocols were approved by the Animal Care and Use Committee of Capital Medical University Beijing Anzhen Hospital (Beijing, China).

### Chronic Intermittent Hypoxia

After 4 weeks of a high-fat diet, mice were randomized into two groups of 10: (1) ApoE^−/−^ mice exposed to normoxia as the control group; (2) ApoE^−/−^ mice exposed to CIH. CIH was performed as previously described by Du et al. (23). Mice were housed in customized standard cages to deliver CIH (Oxycycler A84 BioSpherix, Redfield, NY, USA). Briefly, a gas control system regulated the room airflow (N_2_ and O_2_). A series of programs and flow regulators enabled manipulation of the fraction of inspired O_2_ from 20.9 to 5% over a 120-s period, with a rapid reoxygenation to normal air levels *via* a burst of 100% O_2_ in the succeeding 60-s period. Hypoxic events occurred at a rate of one event per 180 s throughout the 12-h period for 8 weeks. During the other 12-h period, the CIH group was maintained in a normoxic environment. Control mice were exposed to normoxic air at all times with noise and turbulence similar to mice exposed to CIH. All the mice were fed with high-fat diet during the 12-week experiment.

### Tissue Preparation

After 8-week CIH exposure, all mice were starved for 8 h prior to collecting blood samples and were anesthetized with 1% pentobarbital sodium (i.p., 60 mg/kg). Blood samples were collected by puncturing through the cardiac apex and placed at room temperature (25°C) for 2–3 h for sedimentation. After centrifugation at 1,000 × g for 15 min at 4°C, the serum was collected and stored at −80°C for further analysis. After perfusion with natural saline containing 1% heparin, aortas, hearts, and interscapular BAT were dissected. BAT in the interscapular region was a butterfly-shaped tissue with brown color for its rich blood vessels. After it was removed by blunt dissection, it was separated with white adipose tissue and connective tissue according to the color of the tissue by an optical microscope. For Oil Red O staining, the entire aorta extending 5 to 10 mm below the bifurcation of the iliac arteries was removed. Various tissue samples were collected, followed by fixation with 4% paraformaldehyde solution for further histological analysis. A fraction of tissues was frozen in liquid nitrogen for further western blotting and mRNA analysis.

### Histology and Immunohistochemistry

After removal of periarterial connective tissue and fat, the entire mouse aorta was opened and then pinned onto a standard black wax dissection pan using.15 mm-black anodized pins. Aortas were stained in 0.5% Oil Red O (O0625, Sigma-Aldrich, USA) at room temperature for 30 min, followed by washing with 60% isopropanol for 5 min (24).

Mouse arterial tissue and BAT were embedded in paraffin, cut into 5 μm-thick slides. Sections were prepared for hematoxylin and eosin (HE), Masson's trichrome staining, and immunohistochemistry staining. For immunohistochemistry staining, after dewaxing and antigen retrieval with a citrate buffer (pH = 6.0) which was followed by treatment with 3% H_2_O_2_, tissue slides were blocked at room temperature with 3% bovine serum albumin for 60 min (22). Primary antibodies, including CD68 (1:100, ab125212, Abcam, Cambridge, UK), α-SMA (1:100, ab5694, Abcam, Cambridge, UK), and UCP1 (1:100, ab23841, Abcam, Cambridge, UK) were incubated at 4°C overnight. An HRP-conjugated Goat anti-Rabbit IgG (PV-9001, ZSGB-Bio, Beijing, China) secondary antibody was added the next day at room temperature for 1 h. A 3, 30-diaminobenzidine reagent (ZLI-9017, ZSGB-Bio, Beijing, China) was used for color development. The nuclei were counterstained with hematoxylin. Finally, sections were viewed on a Nikon microscope (Japan). Three serial sections were quantified for each sample. Then, another three fields were randomly selected from each slice. The positive area ratio was quantified as positive area divided by the whole area of tissue. Finally, an average value for each specimen was obtained (23).

### Western Blotting

Total proteins from adipose tissues were extracted by a Total Protein Extraction Kit (AT-022, Invent Biotechnologies, USA) (24). Proteins were separated by SDS-PAGE electrophoresis and transferred to a PVDF membrane (Millipore, Germany). After being blocked with 5% fat-free milk in TBST, the membranes were incubated with UCP1 (1:1000, ab23841, Abcam, Cambridge, UK) antibody and β-actin (1:1000, ab213262, Abcam, Cambridge, UK) antibody overnight at 4°C. Subsequently, the membranes were incubated with an infrared Dye 800-conjugated secondary antibodies (1:10,000, 926-32211, LI-COR Biosciences, Lincoln, USA). The images were quantified using the Odyssey infrared imaging system (LI-COR Biosciences, Lincoln, USA) (25). UCP1 protein expression was analyzed with band intensity analysis by Image-Pro Plus 6 (Media Cybernetics, USA).

### RNA Extraction and Quantitative Reverse Transcription-PCR

Ribonucleic acid was extracted using TRIzol reagent (Invitrogen, USA). For reverse transcription, 1 μg of the total RNA was converted to the first-strand cDNA using a reverse transcription kit (RR047A, Takara, Japan). Quantitative reverse transcription-polymerase chain reaction (qRT-PCR) was performed utilizing SYBR Green Master Mix (RR820A, Takara, Japan) on the iCycler iQ system (Bio-Rad, USA). The thermal cycling program was 30 s at 95°C for enzyme activation, 40 cycles of denaturation at 95°C for 5 s, and annealing and extension at 60°C for 30 s. The comparative cycle threshold method was used to determine relative mRNA gene expression as normalized by the GAPDH housekeeping gene. There were three technical replicates for each sample. All primers were synthesized by Qiagen (Beijing, China) as follows:

Angptl4: 5′-GAGGTCCTTCACAGCCTGCA-3′;
5′-TGGGCCACCTTGTGGAAGAG-3′
Slc27a2: 5′-TCGGTTCCTGAGGATACAAGAT-3′;
5′-GGGTCACTTTGCGGTGTTTA-3′
UCP1: 5′-ACTGCCACACCTCCAGTCATT-3′;
5′-CTTTGCCTCACTCAGGATTGG-3′
GAPDH: 5′-CATGGCCTTCCGTGTTCCTA-3′;
5′-GCGGCACGTCAGATCCA-3′

### Serum Analysis

Fasting blood glucose was performed in unanesthetized mice by tail bleeding. Blood glucose was measured with a glucometer.

Triglyceride (TG), total cholesterol (TC), low-density lipoprotein cholesterol (LDL-c), high-density lipoprotein cholesterol (HDL-c) (Roche Diagnostics GmbH, Mannheim, Germany), and free fatty acid (FFA) (DiaSys Diagnostic Systems GmbH, Holzheim, Germany) in serum were measured using commercial kits (26).

The levels of superoxide dismutase (SOD) and glutathione (GSH) in mouse serum were measured using superoxide dismutase assay kit (A001-3, Nanjing Jiancheng Bioengineering Institute, China) and glutathione assay kit (A006-2, Nanjing Jiancheng Bioengineering Institute, China).

### RNA-Sequencing Analysis

Isolated RNA of mouse BAT was used for RNA-seq analysis. cDNA library construction and sequencing were performed by the Wuhan BGI using the BGISEQ-500 platform (China). High-quality reads were aligned to the mouse reference genome using Bowtie2 (China). Expression levels for each of the genes were normalized to fragments per kilobase of exon model per million mapped reads (FPKM) using RNA-seq by Expectation-Maximization (RSEM). Differentially expressed genes (DEGs) were defined by a fold change ≥2 and a false discovery rate (FDR)-corrected *p* < 0.05. Pathway analysis, predominantly based on the Kyoto Encyclopedia of Genes and Genomes (KEGG) database, was used to determine the significant functions and pathways of DEGs. Only pathway categories with an FDR-corrected *p* < 0.05 were considered as significantly enriched (27).

### Statistical Analysis

Continuous data have been presented as mean ± SEM. The normality of values was tested. If values followed a normal distribution, then data were analyzed with student's *t*-test for two independent samples. If not, the Mann-Whitney U test was performed. The statistical significance level for all the tests was set at a two-sided *p* < 0.05. All analyses were performed using SPSS software (SPSS Statistics version 25, IBM Corporation, USA).

## Results

### Basic Characteristics

The body weight of mice remained stable during CIH and did not differ from control conditions (Table 1). Based on the diet-induced obesity in the control group, the effect of CIH on body weight may be blunted. After 8-week CIH exposure, mice of the CIH group had higher levels of blood glucose (Table 1).

**Table 1:** Basic characteristics of mice exposed to CIH or control.

	**Control**	**CIH**	**P**
**Bodyweight (g)**			
Baseline	24.29 ± 0.36	23.32 ± 0.36	0.08
End of the experiment	28.28 ± 0.54	27.22 ± 0.64	0.23
**Blood glucose (mmol/L)**	5.92 ± 0.35	7.98 ± 0.58*	0.01

CIH, chronic intermittent hypoxia; *p < 0.05 vs. control.

### CIH Induced BAT Activation in Atherosclerosis

**Figure 1:**
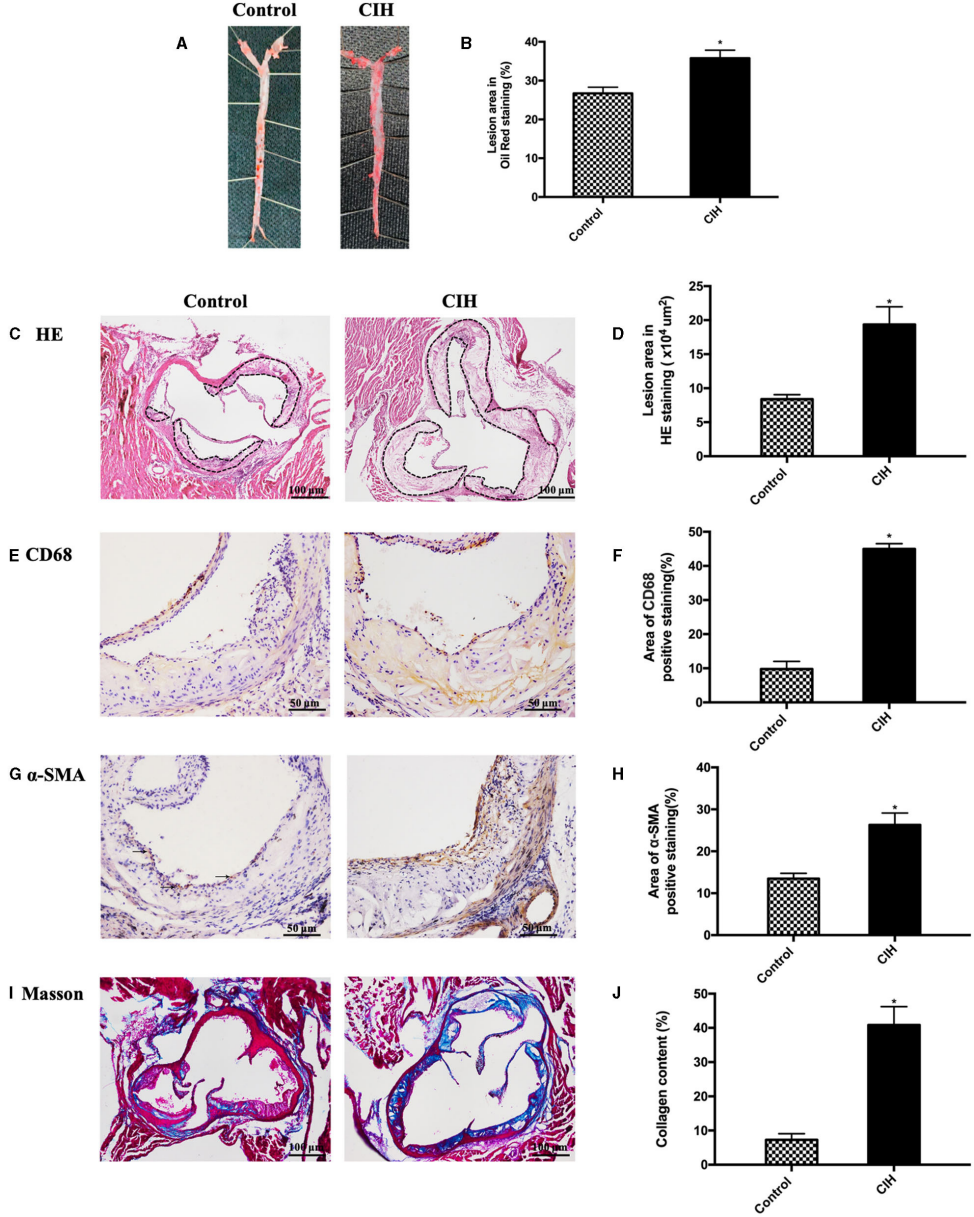
Eight-week exposure of CIH-induced atherosclerosis in ApoE^−/−^ mice. Male ApoE^−/−^ mice exposed to normoxia or CIH grouped as control and CIH group. **(A)** Representative photographs of whole aorta plaque that were stained with Oil Red O. **(B)** Plaque area as a percentage of the total area in Oil Red O staining. **(C)** Representative photographs of plaque in aorta roots that were stained with HE staining. Dashed lines encircle atherosclerotic plaques. **(D)** Lesion area in HE staining. **(E)** Representative photographs of aortic root sections that were immunostained with CD68. **(F)** The quantification of macrophages in aorta plaque. **(G)** Representative photographs of aortic root sections that were immunostained with α-SMA. **(H)** The quantification of smooth muscle cells in aorta plaque. **(I)** Representative photographs of aortic root sections that were stained with Masson. **(J)** The quantification of collagen in aorta plaque. Three serial sections were quantified for each sample. *n* = 7. Data were presented as mean ± SEM. ^*^ CIH vs. control *p* < 0.05.

The study primarily proved that CIH exacerbated atherosclerosis in ApoE^−/−^ mice. We stained the whole aorta with oil red O and the aortic root cross-sections with HE to identify atherosclerotic lesions and found atherosclerotic lesions increased in the CIH group, compared with the control group (Figures 1A–D). Immunohistochemical analysis showed that CD68 positive area, α-SMA positive area, and Masson structures in atherosclerotic plaques were significantly increased after 8 weeks of CIH exposure (Figures 1E–J). These findings showed that CIH accelerated atherosclerosis progressing in ApoE^−/−^ mice, which is consistent with previous findings of the effect of CIH on atherosclerosis (5–7).

**Figure 2:**
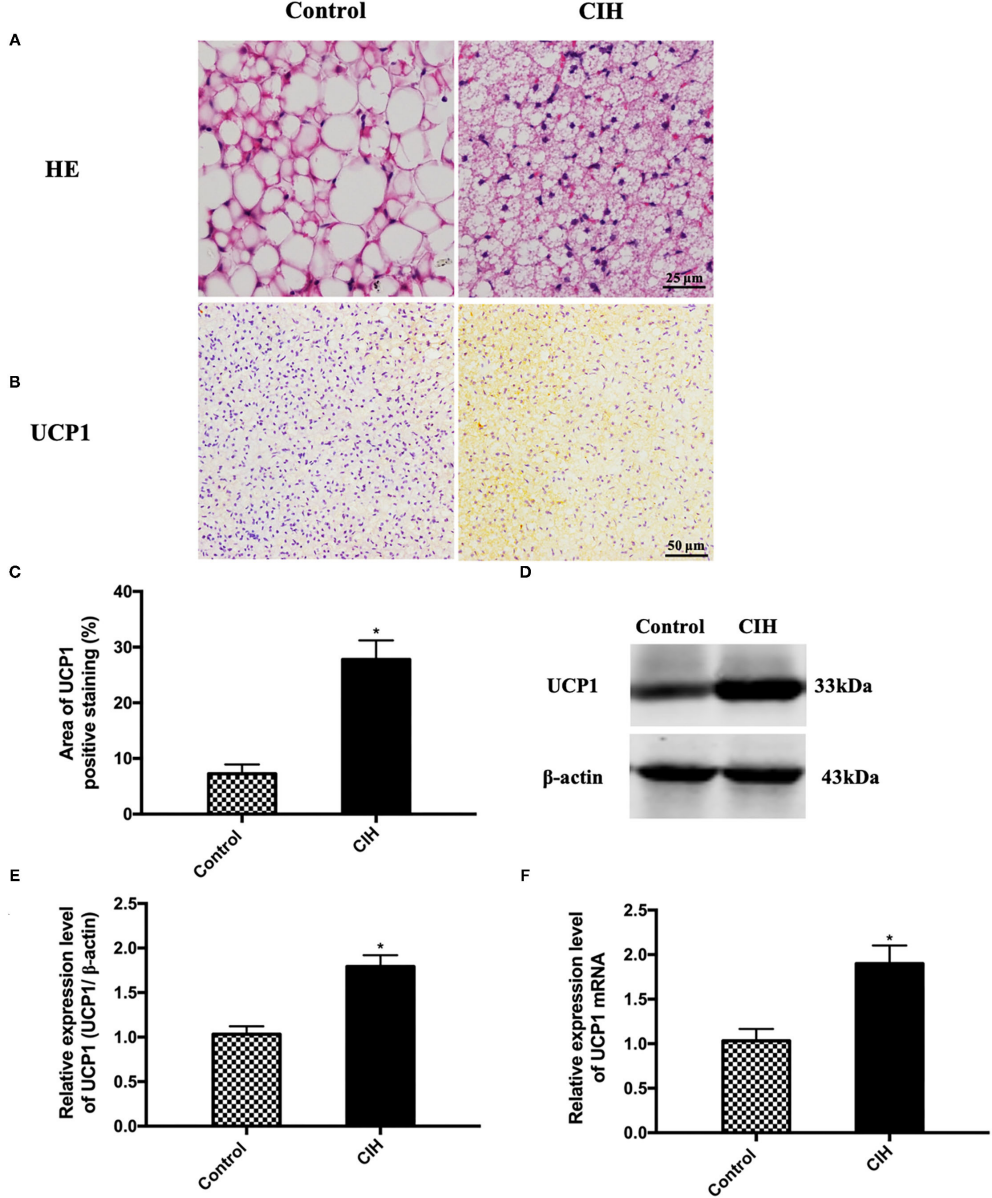
Eight-week exposure of CIH-induced BAT activation in ApoE^−/−^ mice. Male ApoE^−/−^ mice exposed to normoxia or CIH grouped as control and CIH group. **(A)** Representative photographs of BAT that were stained with HE. **(B)** Representative photographs of BAT that were immunostained with UCP1. **(C)** The quantification of UCP1 positive area percentage in BAT. Three serial sections were quantified for each sample. *n* = 7. **(D)** Representative photographs of UCP1 western blotting in BAT. **(E)** Relative expression level of UCP1 in BAT in western blotting. **(F)** UCP1 mRNA expression in BAT. *n* = 5. Data were presented as mean ± SEM. * CIH vs. control *p* < 0.05.

Afterward, we made a histological analysis of BAT and found an activated phenotype in CIH-treated BAT. BAT HE staining showed that the adipocyte sizes were smaller in CIH-exposed adipose tissues compared with controls (Figure 2A). This histological analysis supported an activated phenotype in CIH-exposed BAT. Immunohistochemical analysis showed that the UCP1 positive area of BAT was increased in the CIH group (Figures 2B,C). Western blotting also showed higher UCP1 protein levels in the BAT of the CIH group (Figures 2D,E). Consistently, the UCP1 mRNA level also significantly increased in the BAT of the CIH group (Figure 2F). All these data indicate that CIH exposure increased the expression level of UCP1 which is the hallmark of BAT activation. These findings showed that CIH augments BAT activation during the process of atherosclerosis formation.

### Activation of Lipolysis by CIH Exposure

**Figure 3:**
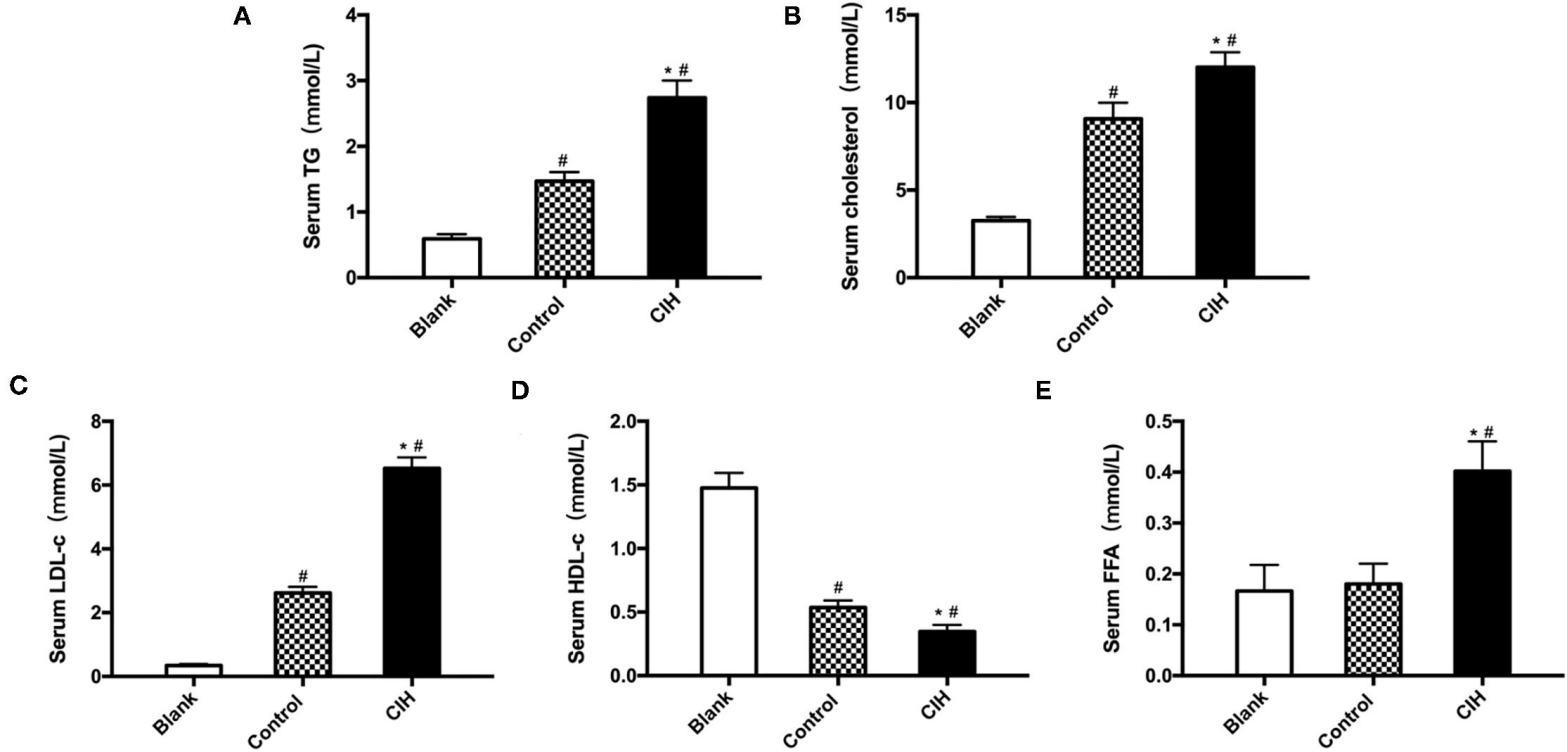
Hyperlipidemia and lipolysis induced by CIH. Male wild-type C57BL/6J mice as Blank group. Male ApoE^−/−^ mice exposed to normoxia or CIH grouped as control and CIH group. **(A)** TG levels in the serum of mice. **(B)** TC levels in the serum of mice. **(C)** LDL-c levels in the serum of mice. **(D)** HDL-c levels in the serum of mice. **(E)** FFA levels in the serum of mice. *n* = 6. Data were presented as mean ± SEM. * CIH vs. control *p* < 0.05. ^#^ vs. Blank *p* < 0.05.

Atherosclerotic plaque growth was associated with the disturbance of lipid metabolism and UCP1 has been reported involved in lipid metabolism. Due to this, we measured blood lipid levels. In ApoE^−/−^ mice, exposure to CIH elevated serum levels of TG, TC, and LDL-c (Figures 3A–C). Serum levels of HDL-c were decreased (Figure 3D). Serum levels of FFA were significantly decreased because of an active phenotype of lipolysis (Figure 3E). Compared with wild-type C57BL/6J mice, both control and CIH mice showed the disturbance of lipid indicating that obesity and CIH can lead to increase adipose tissue lipolysis. These findings indicated that UCP1- mediated lipolysis is involved in CIH-induced atherosclerosis.

### Alteration of Expression Levels of Lipolysis-Associated Gene Products

**Figure 4:**
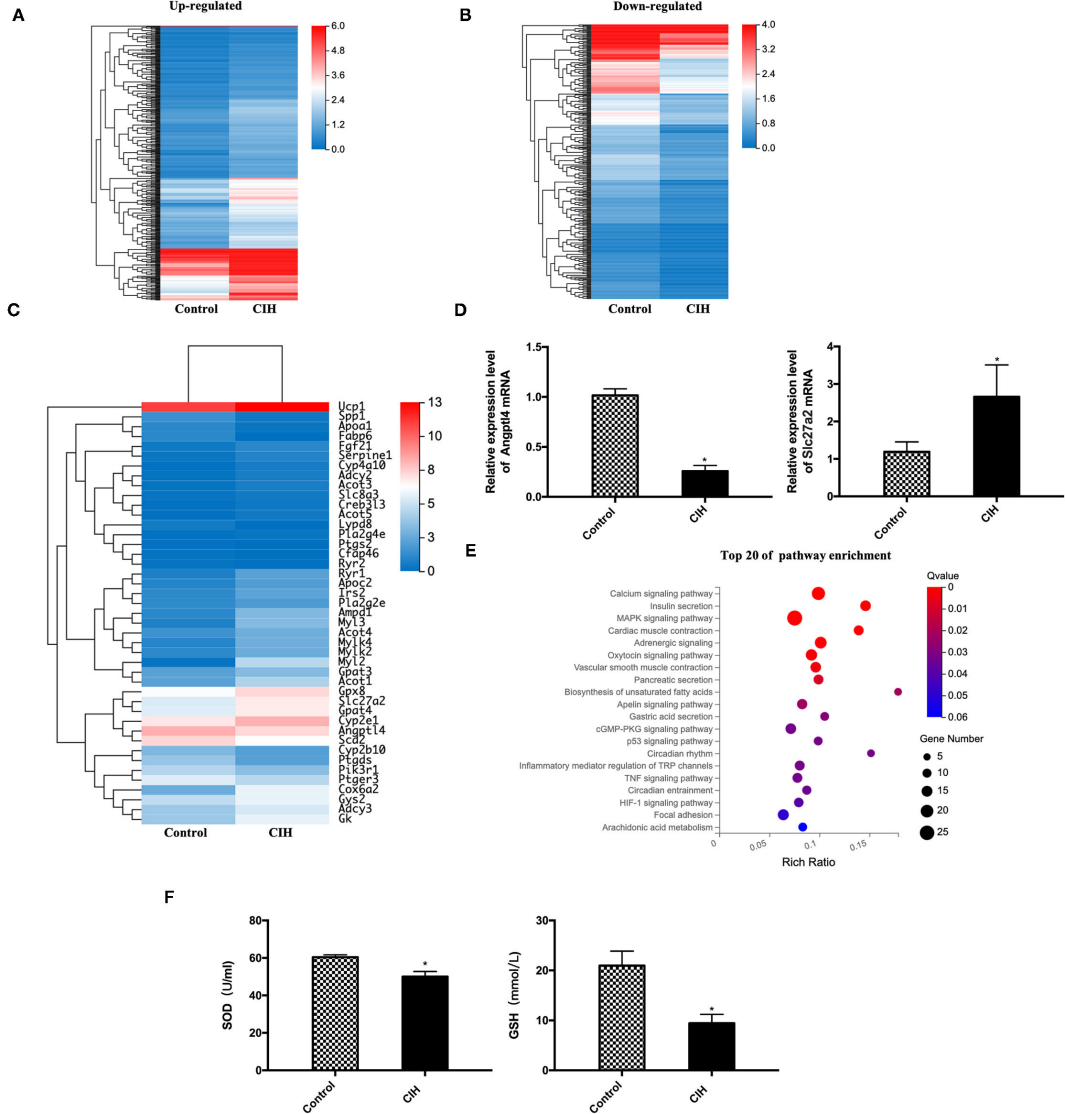
Alteration of gene expression profiling and the pathway enrichment in ApoE^−/−^ mouse BAT exposed to CIH. Male ApoE^−/−^ mice exposed to normoxia or CIH grouped as control and CIH group. **(A)** A total of 387 up-regulated genes in BAT of CIH group compared with the control group. **(B)** A total of 337 down-regulated genes in BAT of CIH group compared with the control group. **(C)** A total of 43 lipolysis and lipid metabolism-associated mRNA of BAT showed different expression profiling between the CIH and control group. **(D)** Angptl4 and Slc27a2 mRNA expression in BAT. *n* = 6. Data were presented as mean ± SEM. * CIH versus control *p* < 0.05. **(E)** The top 20 of pathway enrichment based on KEGG was classified according to their functions related to metabolism, lipolysis, and signaling pathways. Calcium signaling pathway (with 20 genes), MAPK signaling pathway (with 25 genes), and adrenergic signaling pathway (with 16 genes) include the most gene number among the significantly enriched pathways. KEGG indicates Kyoto Encyclopedia of Genes and Genomes. *n* = 3. **(F)** SOD levels and GSH levels in mouse serum. *n* = 6. Data were presented as mean ± SEM. * CIH vs. control *p* < 0.05. SOD indicates superoxide dismutase. GSH indicates glutathione.

To explore molecular mechanisms, whole mRNA expression profiling in BAT was analyzed. CIH exposure resulted in noticeable alterations of gene expression patterns with 387 up- and 337 down-regulation of genes (Figures 4A,B). Among them, a nearly three-fold increase of UCP1 expression was detected in CIH-treated BAT compared with the control group. Interestingly, 43 lipolysis and lipid metabolism-associated mRNA of BAT showed different expression profiling between the CIH and control group (Figure 4C). Among them, we validated the genes which play important role in regulating lipoprotein metabolism by RT-PCR. CIH exposure decreased the expression of Angptl4 mRNA in BAT and increased the expression of Slc27a2 mRNA in BAT (Figure 4D).

Then the top 20 of pathway enrichment was classified according to their functions related to metabolism, lipolysis, and signaling pathways (Figure 4E). Calcium signaling pathway (with 20 genes), MAPK signaling pathway (with 25 genes), and adrenergic signaling pathway (with 16 genes) include the most gene number among the significantly enriched pathways. These data showed that CIH significantly modulated the cellular and molecular components in the BAT microenvironment.

Owing to UCP1 has been reported to induce mitochondria reactive oxygen species (ROS). We measured the production of antioxidant markers such as SOD and GSH of mouse serum. CIH exposure decreased the expression level of SOD and GSH (Figure 4F), indicating that oxidative stress also participating in the process of CIH-induced atherosclerosis and it may act with UCP1.

## Discussion

In this work, we provided new insights into the active phenotype of BAT in CIH-induced atherosclerosis and demonstrated that UCP1- mediated lipolysis is involved in this process. First, by using a CIH-induced atherosclerosis mouse model, we demonstrated that BAT is activated with upregulated UCP1. Meanwhile, the disturbance of lipid metabolism and lipolysis were observed along with BAT activation. Finally, we explored the possible molecular mechanisms and pathways of BAT activation which are involved in CIH-induced atherosclerosis. In summary, our data provided novel evidence that BAT activation is involved in CIH-induced atherosclerosis by regulating lipolysis.

Chronic intermittent hypoxia has been widely known as an independent risk factor for atherosclerosis concerning numerous mechanisms including inflammation, oxidative stress, and blood lipid distribution (28). In clinical, it has been reported that OSA is associated with hypercholesterolemia, independent of adiposity, and partially reversible with continuous positive airway pressure therapy, even with no changes in body weight (29, 30). In the animal model, CIH can up-regulate the sterol regulatory element-binding protein-1 (SREBP-1) and stearoyl-CoA desaturase-1 (SCD-1), increase the level of cholesterol esters and triglycerides (7, 31). In our study, we observed the increased level of serum TC, TG, and LDL-c in ApoE^−/−^ mice exposed to CIH, accompanied by accelerated atherosclerosis. These findings all supported that CIH plays a major role in the pathogenesis of the dysregulation of lipid metabolism. However, the precise mechanism by which CIH induces dyslipidemia is not well understood.

Brown adipose tissue is the main site of adaptive thermogenesis. Brown adipocytes contain many mitochondria with a high oxidative capacity and have UCP1 in their inner membrane. UCP1, which is only expressed in brown adipocytes, is a biomarker of BAT activation (18). Each brown adipocyte is in close proximity to a nerve ending that releases norepinephrine on sympathetic stimulation (32). Norepinephrine subsequently binds to the β3- adrenergic receptor on the membrane of the brown adipocyte (33). It is known that the sympathetic system is over-activated in CIH leading to an increased level of norepinephrine, which acts on the β3- adrenergic receptors (10, 11). However, whether the activated sympathetic system can affect BAT activation in CIH has not been explored. Then in our study, after 8-week CIH exposure, we found the activation of β3- adrenergic receptors in BAT (Supplementary Figure 1), and the level of UCP1 protein and mRNA was increased in BAT. The present study demonstrated that BAT is activated in CIH-induced atherosclerosis.

Upon BAT activation, intracellular lipolysis causes depletion of intracellular triglyceride stores that subsequently need to be replenished *via* de novo lipogenesis and *via* the uptake of FA from the circulation, mainly *via* selective delipidation of TG-rich lipoproteins (TRLs) through the hydrolyzing action of lipoprotein lipase (LPL). The released FA are taken up by cluster of differentiation 36 (CD36) and FA transport proteins (FATP) and stored in the lipid droplets as TG again in brown adipocyte (34). Then the activation of protein kinase A (PKA) in brown adipocytes enhances the activity of lipolytic enzymes that are associated with the intracellular lipid droplets, leading to the release of FA that enters the mitochondria for β-oxidation. In this study, we found the mRNA level of angiopoietin-like protein 4 (Angptl4), a potent inhibitor of the LPL activity (35), was downregulated and Slc27a2, the subunit of FATP, was upregulated in BAT of the CIH group. These all indicated that in CIH BAT was prolonged activated and affected lipolysis.

We further explored the function of BAT activation in CIH-induced atherosclerosis. Berbée et al. (36) proved that BAT activation increases lipolytic processing and hepatic clearance of lipoproteins, ameliorates dyslipidemia, and prevents atherosclerosis in E3L.CETP mice. However, BAT activation did not protect ApoE^−/−^ and Ldlr^−/−^ mice from atherosclerosis (36). Conversely, the study of Sui et al. reported that β3-adrenergic receptor agonist activated BAT and increase plasma levels of both LDL-c and very LDL-c remnants in ApoE^−/−^ mice. This phenomenon is dependent on thermogenesis-triggered lipolysis. Genetic deletion of UCP1 completely abrogates dyslipidemia (24). Meanwhile, in various cold environments, BAT activation also has different effects on atherosclerosis (21). All these studies suggested that BAT activation owns complex functions. The study of Dong et al. reported that BAT activation in 4°C promoted lipolysis and atherosclerosis in ApoE^−/−^ mice and Ldlr^−/−^ mice. Deletion of UCP1 completely protected mice from cold-induced atherosclerotic lesions (22). This contradictory phenomenon has been explained by the study of Hoeke G et al. because an intact apoE-LDLR pathway is crucial for the cholesterol-lowering, wherein after BAT activation, the lipid metabolism mode of ApoE^−/−^ and Ldlr^−/−^ mice would be different from that of E3L.CETP mice (37). In the present study, we also found that serum TC, TG, LDL-c, and FFA were increased, accompanied by BAT activation in ApoE^−/−^ mice exposed to CIH. RNA-Sequencing analysis revealed that 43 lipolysis and lipid metabolism-associated mRNA of BAT were affected by CIH. These data supported that regulating lipolysis may be one of the mechanisms in which BAT activation affects atherosclerosis in CIH.

Finally, we further analyzed the DEGs between the control group and the CIH group and determine the significant functions and pathways of DEGs by KEGG. The calcium signaling pathway, MAPK signaling pathway, and adrenergic signaling pathway include the most gene number among the significantly enriched pathways. It has been reported that calcium overload on mitochondria can induce brown adipose dysfunction (38). P38-MAPK and adrenergic pathway have been reported involved in BAT activation in a previous study (37, 39). This results in the activation of adenylyl cyclase to produce cyclic AMP that activates PKA to phosphorylate the lipolytic enzymes adipose triglyceride lipase, hormone-sensitive lipase, and monoglycerol lipase, leading to increased intracellular lipolysis. FA that is released subsequently enter the mitochondria where they are broken down into substrates for the citric acid cycle, leading to activation of the electron transport chain and uncoupled respiration through UCP1 that results in the generation of heat instead of ATP (12). These data indicated that the calcium signaling pathway, MAPK signaling pathway, and adrenergic signaling pathway play the most important role in CIH-induced BAT activation.

The present study has several limitations. First, we observed BAT activation accompanied by lipolysis in CIH. Though UCP1 has been recognized as the biomarker of BAT activation, it is still an indirect indicator. Additionally, the mechanism of how BAT activation contributed to lipolysis in CIH was poorly understood. Other physiological processes which were associated with lipolysis during BAT activation, such as hepatic cholesterol synthesis and taking up of glucose in brown adipocytes, need to be further explored. Therefore, further investigation is needed to confirm the DEGs which were found by RNA-Sequencing regulate BAT activation and lipolysis in CIH. Second, we speculated other pathways may be involved in the BAT activation in CIH by KEGG analysis. In the future, these pathways need further investigation.

In conclusion, this study first demonstrated that BAT activation is involved in the progression of CIH-induced atherosclerosis, possibly by stimulating lipolysis.

## Data Availability Statement

The datasets presented in this study can be found in online repositories. The names of the repository/repositories and accession number(s) can be found in the article/Supplementary Material.

## Ethics Statement

The animal study was reviewed and approved by Animal Care and Use Committee of Capital Medical University Beijing Anzhen Hospital (Beijing, China).

## Author Contributions

YW (first author) is a doctorial candidate of grade 2020; YW (fifth author) is a doctorial candidate of grade 2019. YW (first author) performed experiments, analyzed and interpreted the data, and drafted the manuscript. H-fJ contributed resources and secured funding, designed research, and conducted the experiments. B-bL, L-lC, YW (fifth author), and X-yL conducted the experiments. MS analyzed and interpreted the data and edited the paper. X-fW contributed resources and secured funding, designed research, supervised the project, and reviewed the manuscript. All authors contributed to the article and approved the submitted version.

## Funding

This work was supported by Beijing Natural Science Foundation and Municipal Education Commission Grant KZ202010025045, the National Natural Science Foundation of China (NSFC) Grants 82071573, 81470492, and 81670317), Beijing Talents Project 2020A39 to X-fW. National Natural Science Foundation of China (NSFC) Grant 81970392 and Beijing Natural Science Foundation J190010 to H-fJ.

## Conflict of Interest

The authors declare that the research was conducted in the absence of any commercial or financial relationships that could be construed as a potential conflict of interest.

## Publisher's Note

All claims expressed in this article are solely those of the authors and do not necessarily represent those of their affiliated organizations, or those of the publisher, the editors and the reviewers. Any product that may be evaluated in this article, or claim that may be made by its manufacturer, is not guaranteed or endorsed by the publisher.

## References

[1] Herrington W Lacey B Sherliker P Armitage J Lewington S. Epidemiology of atherosclerosis and the potential to reduce the global burden of atherothrombotic disease. Circ Res. (2016) 118:535–46. 10.1161/CIRCRESAHA.115.307611 26892956

[2] Peppard PE Young T Barnet JH Palta M Hagen EW Hla KM. Increased prevalence of sleep-disordered breathing in adults. Am J Epidemiol. (2013) 177:1006–14. 10.1093/aje/kws342 23589584 PMC3639722

[3] Javaheri S Barbe F Campos-Rodriguez F Dempsey JA Khayat R Javaheri S. Sleep apnea: types, mechanisms, and clinical cardiovascular consequences. J Am Coll Cardiol. (2017) 69:841–58. 10.1016/j.jacc.2016.11.069 28209226 PMC5393905

[4] Gottlieb DJ Punjabi NM. Diagnosis and management of obstructive sleep apnea: a review. JAMA. (2020) 323:1389–400. 10.1001/jama.2020.3514 32286648

[5] Song D Fang G Greenberg H Liu SF. Chronic intermittent hypoxia exposure-induced atherosclerosis: a brief review. Immunol Res. (2015) 63:121–30. 10.1007/s12026-015-8703-8 26407987

[6] Lin H Zeng Y Wang Z. Maternal chronic intermittent hypoxia in rats causes early atherosclerosis with increased expression of Caveolin-1 in offspring. Sleep Breath. (2019) 23:1071–77. 10.1007/s11325-019-01781-y 30685852

[7] Savransky V Nanayakkara A Li J Bevans S Smith PL Rodriguez A. Chronic intermittent hypoxia induces atherosclerosis. Am J Respir Crit Care Med. (2007) 175:1290–7. 10.1164/rccm.200612-1771OC 17332479 PMC2176090

[8] Song D Fang G Mao SZ Ye X Liu G Miller EJ. Selective inhibition of endothelial NF-κB signaling attenuates chronic intermittent hypoxia-induced atherosclerosis in mice. Atherosclerosis. (2018) 270:68–75. 10.1016/j.atherosclerosis.2018.01.027 29407890

[9] Ma L Zhang J Liu Y. Roles and mechanisms of obstructive sleep apnea-hypopnea syndrome and chronic intermittent hypoxia in atherosclerosis: evidence and prospective. Oxid Med Cell Longev. (2016) 2016:8215082. 10.1155/2016/8215082 27293515 PMC4884866

[10] Rukhadze I Fenik VB Benincasa KE Price A Kubin L. Chronic intermittent hypoxia alters density of aminergic terminals and receptors in the hypoglossal motor nucleus. Am J Respir Crit Care Med. (2010) 182:1321–9. 10.1164/rccm.200912-1884OC 20622040 PMC3001268

[11] Hui AS Striet JB Gudelsky G Soukhova GK Gozal E Beitner-Johnson D. Regulation of catecholamines by sustained and intermittent hypoxia in neuroendocrine cells and sympathetic neurons. Hypertension. (2003) 42:1130–6. 10.1161/01.HYP.0000101691.12358.26 14597643

[12] Cannon B Nedergaard J. Brown adipose tissue: function and physiological significance. Physiol Rev. (2004) 84:277–359. 10.1152/physrev.00015.2003 14715917

[13] Von Bank H Hurtado-Thiele M Oshimura N Simcox J. Mitochondrial lipid signaling and adaptive thermogenesis. Metabolites. (2021) 11:124. 10.3390/metabo11020124 33671745 PMC7926967

[14] Porter C Herndon DN Chondronikola M Chao T Annamalai P Bhattarai N. Human and mouse brown adipose tissue mitochondria have comparable UCP1 function. Cell Metab. (2016) 24:246–55. 10.1016/j.cmet.2016.07.004 27508873 PMC5201422

[15] Asano A Kimura K Saito M. Cold-induced mRNA expression of angiogenic factors in rat brown adipose tissue. J Vet Med Sci. (1999) 61:403–9. 10.1292/jvms.61.403 10342292

[16] Lim S Honek J Xue Y Seki T Cao Z Andersson P. Cold-induced activation of brown adipose tissue and adipose angiogenesis in mice. Nat Protoc. (2012) 7:606–15. 10.1038/nprot.2012.013 22383039

[17] Brychta RJ Chen KY. Cold-induced thermogenesis in humans. Eur J Clin Nutr. (2017) 71:345–52. 10.1038/ejcn.2016.223 27876809 PMC6449850

[18] Villarroya F Cereijo R Villarroya J Giralt M. Brown adipose tissue as a secretory organ. Nat Rev Endocrinol. (2017) 13:26–35. 10.1038/nrendo.2016.136 27616452

[19] Stanford KI Middelbeek RJ Townsend KL Lee MY Takahashi H So K. A novel role for subcutaneous adipose tissue in exercise-induced improvements in glucose homeostasis. Diabetes. (2015) 64:2002–14. 10.2337/db14-0704 25605808 PMC4439563

[20] Betz MJ Enerbäck S. Human brown adipose tissue: what we have learned so far. Diabetes. (2015) 64:2352–60. 10.2337/db15-0146 26050667

[21] Kataoka N Takeuchi T Kusudo T Li Y Endo Y Yamashita H. Lack of UCP1 stimulates fatty liver but mediates UCP1-independent action of beige fat to improve hyperlipidemia in Apoe knockout mice. Biochim Biophys Acta Mol Basis Dis. (2020) 1866:165762. 10.1016/j.bbadis.2020.165762 32179129

[22] Dong M Yang X Lim S Cao Z Honek J Lu H. Cold exposure promotes atherosclerotic plaque growth and instability via UCP1-dependent lipolysis. Cell Metab. (2013) 18:118–29. 10.1016/j.cmet.2013.06.003 23823482 PMC3701322

[23] Du Y Wang X Li L Hao W Zhang H Li Y. miRNA-mediated suppression of a cardioprotective cardiokine as a novel mechanism exacerbating post-MI remodeling by sleep breathing disorders. Circ Res. (2020) 126:212–28. 10.1161/CIRCRESAHA.119.315067 31694459

[24] Sui W Li H Yang Y Jing X Xue F Cheng J. Bladder drug mirabegron exacerbates atherosclerosis through activation of brown fat-mediated lipolysis. Proc Natl Acad. (2019) 116:10937–42. 10.1073/pnas.1901655116 31085638 PMC6561204

[25] Li Y Chen B Yang X Zhang C Jiao Y Li P. S100a8/a9 signaling causes mitochondrial dysfunction and cardiomyocyte death in response to ischemic/reperfusion injury. Circulation. (2019) 140:751–64. 10.1161/CIRCULATIONAHA.118.039262 31220942

[26] Miida T Nishimura K Okamura T. Validation of homogeneous assays for HDL-cholesterol using fresh samples from healthy and diseased subjects. Atherosclerosis. (2014) 233:253–9. 10.1016/j.atherosclerosis.2013.12.033 24529153

[27] Love MI Huber W Anders S. Moderated estimation of fold change and dispersion for RNA-seq data with DESeq2. Genome Biol. (2014) 15:550. 10.1186/s13059-014-0550-8 25516281 PMC4302049

[28] Drager LF McEvoy RD Barbe F Lorenzi-Filho G Redline S. INCOSACT Initiative (International Collaboration of Sleep Apnea Cardiovascular Trialists): sleep apnea and cardiovascular disease: lessons from recent trials and need for team science *Circulation*. (2017) 136:1840–50. 10.1161/CIRCULATIONAHA.117.029400 29109195 PMC5689452

[29] Robinson GV Pepperell JC Segal HC Davies RJ Stradling JR. Circulating cardiovascular risk factors in obstructive sleep apnoea: data from randomised controlled trials. Thorax. (2004) 59:777–82. 10.1136/thx.2003.018739 15333855 PMC1747125

[30] Chin K Shimizu K Nakamura T Narai N Masuzaki H Ogawa Y. Changes in intra-abdominal visceral fat and serum leptin levels in patients with obstructive sleep apnea syndrome following nasal continuous positive airway pressure therapy. Circulation. (1999) 100:706–12. 10.1161/01.CIR.100.7.706 10449691

[31] Li J Grigoryev DN Ye SQ Thorne L Schwartz AR Smith PL. Chronic intermittent hypoxia upregulates genes of lipid biosynthesis in obese mice. J Appl Physiol. (2005) 99:1643–8. 10.1152/japplphysiol.00522.2005 16037401

[32] Cypess AM Weiner LS Roberts-Toler C Franquet Elía E Kessler SH Kahn PA. Activation of human brown adipose tissue by a β3-adrenergic receptor agonist. Cell Metab. (2015) 21:33–8. 10.1016/j.cmet.2014.12.009 25565203 PMC4298351

[33] Townsend KL Tseng YH. Brown fat fuel utilization and thermogenesis. Trends Endocrinol Metab. (2014) 25:168–177. 10.1016/j.tem.2013.12.004 24389130 PMC3972344

[34] Bartelt A Bruns OT Reimer R Hohenberg H Ittrich H Peldschus K. Brown adipose tissue activity controls triglyceride clearance. Nat Med. (2011) 17:200–5. 10.1038/nm.2297 21258337

[35] Drager LF Li J Shin MK Reinke C Aggarwal NR Jun JC. Intermittent hypoxia inhibits clearance of triglyceride-rich lipoproteins and inactivates adipose lipoprotein lipase in a mouse model of sleep apnoea. Eur Heart J. (2012) 33:783–90. 10.1093/eurheartj/ehr097 21478490 PMC3303712

[36] Berbée JF Boon MR Khedoe PP Bartelt A Schlein C Worthmann A. Brown fat activation reduces hypercholesterolaemia and protects from atherosclerosis development. Nat Commun. (2015) 6:6356. 10.1038/ncomms7356 25754609 PMC4366535

[37] Hoeke G Kooijman S Boon MR Rensen PC Berbée JF. Role of brown fat in lipoprotein metabolism and atherosclerosis. Circ Res. (2016) 118:173–82. 10.1161/CIRCRESAHA.115.306647 26837747

[38] Kazak L Chouchani ET Stavrovskaya IG Lu GZ Jedrychowski MP Egan DF. UCP1 deficiency causes brown fat respiratory chain depletion and sensitizes mitochondria to calcium overload-induced dysfunction. Proc Natl Acad Sci USA. (2017) 114:7981–6. 10.1073/pnas.1705406114 28630339 PMC5544316

[39] Bordicchia M Liu D Amri EZ Ailhaud G Dessì-Fulgheri P Zhang C. Cardiac natriuretic peptides act via p38 MAPK to induce the brown fat thermogenic program in mouse and human adipocytes. J Clin Invest. (2012) 122:1022–36. 10.1172/JCI59701 22307324 PMC3287224

