# Supplementary figures and images for: Brown Adipose Tissue Activation Is Involved in Atherosclerosis of ApoE−/− Mice Induced by Chronic Intermittent Hypoxia
Source: Front Cardiovasc Med. 2021 Oct 26;8:751519. doi: 10.3389/fcvm.2021.751519 (PMC8576199; doi:10.3389/fcvm.2021.751519)

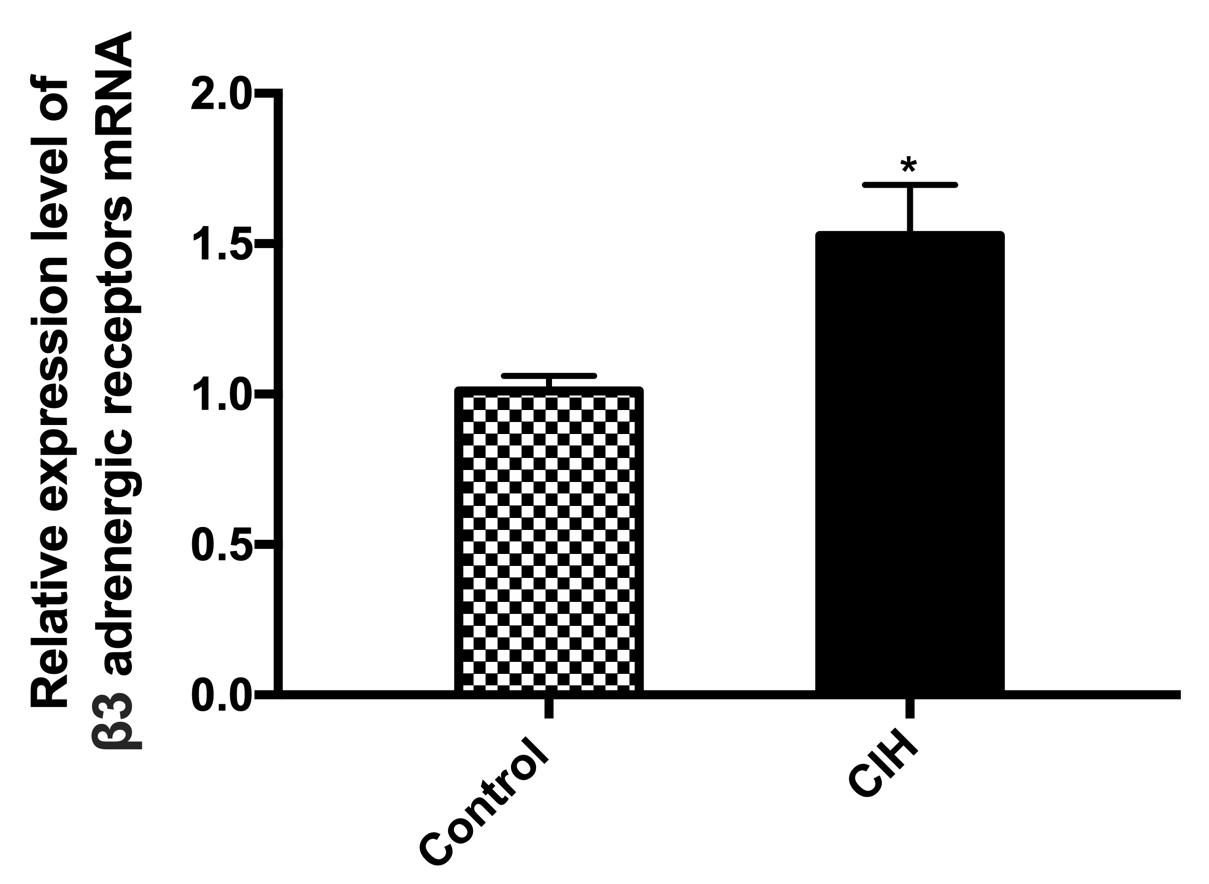

Supplement: Supplementary file 1 [file Image_1.TIFF]
